# Bladder cancer detection by urinary extracellular vesicle mRNA analysis

**DOI:** 10.18632/oncotarget.25998

**Published:** 2018-08-28

**Authors:** Taku Murakami, Cindy M. Yamamoto, Tomoshige Akino, Hiroshi Tanaka, Nobuyuki Fukuzawa, Hidetaka Suzuki, Takahiro Osawa, Takahiro Tsuji, Toshimori Seki, Hiroshi Harada

**Affiliations:** ^1^ Hitachi Chemical Co. America, Ltd., Irvine, CA, USA; ^2^ Sapporo City General Hospital, Sapporo, Japan; ^3^ Graduate School of Medicine, Hokkaido University, Sapporo, Japan

**Keywords:** bladder cancer, biomarker, extracellular vesicles, exosome, mRNA

## Abstract

**Objective:**

Urinary extracellular vesicles (EV) could be promising biomarkers for urological diseases. In this retrospective feasibility study, we conducted biomarker screening for early stage bladder cancer using EV mRNA analysis.

**Methods:**

Biomarker candidates were identified through RNA-seq analysis of urinary EV from patients with non-muscle invasive bladder cancer (N=3), advanced urothelial cancer (N=3), no residual tumor after TURBT (N=2), and healthy and disease controls (N=4). Diagnostic performance was evaluated by RT-qPCR in a larger patient group including bladder cancer (N=173), renal pelvis and ureter cancer (N=33), no residual tumor and non-cancer disease control (N=36).

**Results:**

Urinary EV *SLC2A1*, *GPRC5A* and *KRT17* were overexpressed in pT1 and higher stage bladder cancer by 20.6-fold, 18.2-fold and 29.5-fold, respectively. These genes allowed detection of non-muscle invasive bladder cancer (AUC: 0.56 to 0.64 for pTa, 0.62 to 0.80 for pTis, and 0.82 to 0.86 for pT1) as well as pT2 and higher muscle invasive bladder cancer (AUC: 0.72 to 0.90). Subgroup analysis indicated that these markers could be useful for the detection of cytology-negative/-suspicious and recurrent bladder cancers.

**Conclusion:**

Three urinary EV mRNA were discovered to be elevated in bladder cancer. Urinary EV mRNA are promising biomarkers of urothelial cancer and worth further investigation.

## INTRODUCTION

National Cancer Institute estimated that there will be approximately 74,000 new bladder cancer cases and 14,000 deaths in the United States alone [1]. About 75% of bladder cancer is non-muscle-invasive cancer (Ta, Tis and T1) and about 25% is muscle-invasive cancer (T2, T3 and T4) [1]. Since the recurrence and progression rate is 50 to 70% for the non-muscle-invasive cancers, the patients with bladder cancer history require lifelong monitoring of recurrence, which makes bladder cancer the most expensive cancer from diagnosis to treatment in the US [2]. Other urothelial cancers located in ureters and renal pelvises are rare compared to bladder cancer, however 20% to 50% of the patients will have bladder cancer in the future [3]. The recurrent nature of urothelial cancers demands non-invasive diagnostic tools for follow-up of patients.

Current gold standard of bladder cancer detection is cystoscopy with urine cytology. The sensitivity and specificity of cystoscopy is 87% and 100%, respectively [1]. Due to the invasiveness of cystoscopy, several urinary markers have been proposed however none of the existing markers was validated yet to replace cystoscopy [1]. Urine cytology has the specificity of 96%, however the sensitivity is only 44% and even lower (4 to 31%) for low-grade tumors [4]. Other urinary markers were evaluated in clinical studies and approved by the Food and Drug Administration, such as bladder tumor antigen and nuclear matrix protein 22 [4]. These diagnostics show similar or better performances to urine cytology however still not satisfactory especially for low stage and low grade tumors [4, 5], therefore new non-invasive biomarkers especially with higher sensitivity are in need.

Extracellular vesicles (EV) are known to be released into the urinary space from all the areas of the nephrons by encapsulating the cytoplasmic molecules of the cell of origin [6]. Several studies showed that tumors generates larger EV at higher concentrations [7] and tumor-derived EV mediates tumor development and progression. EV from muscle invasive bladder tumor has been shown to cause epithelial-to-mesenchymal transition of urothelial cells [8]. Urinary EV RNA-seq analysis of a healthy volunteer indicated not only kidney specific genes but also bladder specific genes (unpublished data). Since urothelial cancers are located on the urothelium and directly in contact with urine, it is highly possible that EV originating from urothelial cancers are released into urine, suggesting that urinary EV could be a source of urothelial cancer biomarkers. Urinary EV are released not only from tumors but also from normal and injured cells therefore it may be expected that molecular signatures of urothelial cancer could be obtained not only from the tumors but also from injured peripheral tissues.

Our previous studies employed an EV mRNA assay to conduct biomarker screenings for hematologic, kidney and ovarian disorders, where the assay performance such as sensitivity, linearity, and reproducibility was optimized and characterized [9–12]. In this retrospective feasibility study for bladder cancer, our objectives were to conduct a biomarker screening of bladder cancer using urine samples from urothelial cancer patients with various grades and stages and to evaluate diagnostic accuracy of the marker candidates compared to the conventional urine cytology and other assays.

## RESULTS

### Urinary EV RNA-seq analysis for marker screening

**Figure 1:**
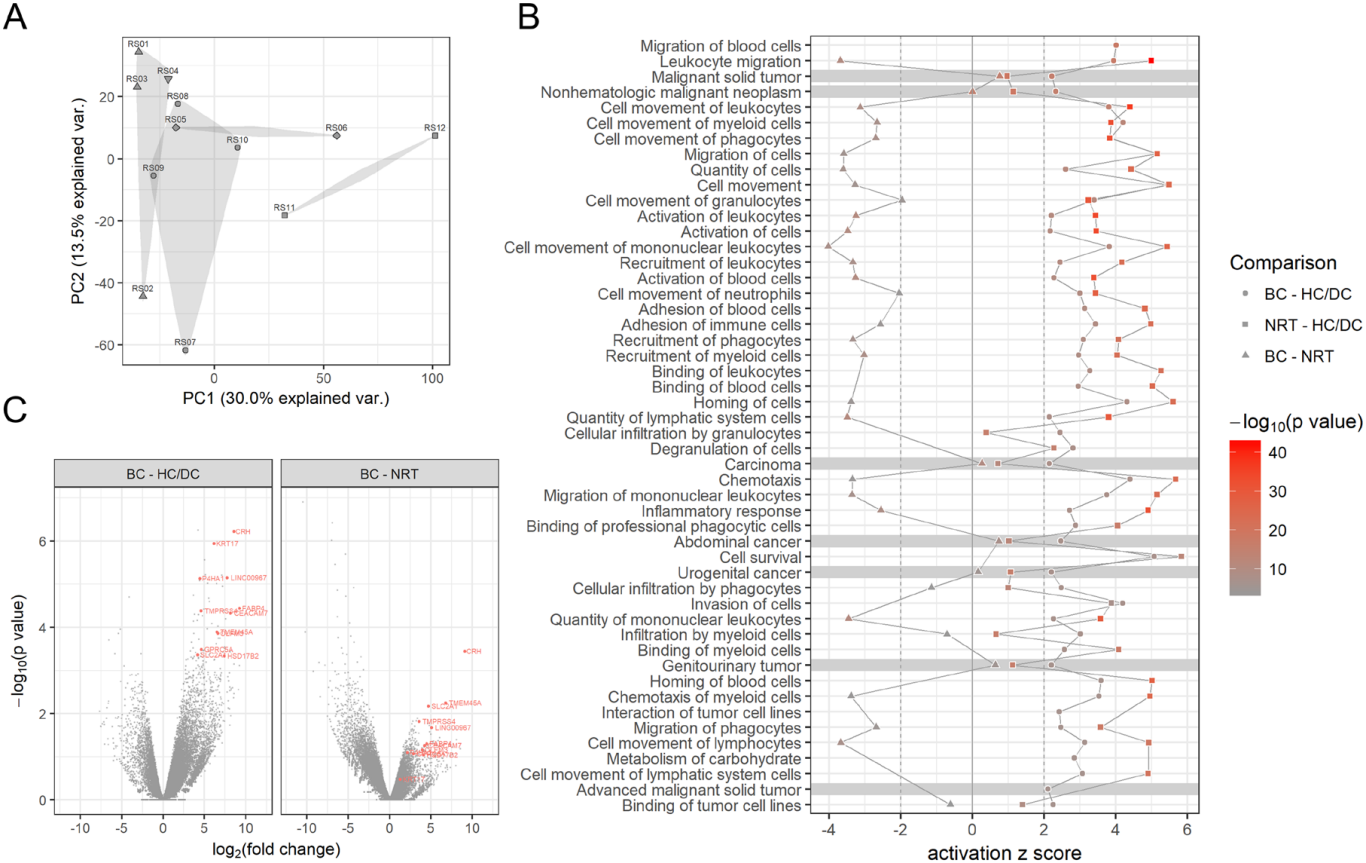
RNA-seq analysis of urinary EV mRNA **(A)** Unsupervised clustering analysis. Unsupervised clustering analysis of urinary EV mRNA profiles showed different clusters corresponding to healthy and disease controls (HC/DC, triangles and inverted triangle, respectively), no residual tumor (NRT, rhombuses), bladder cancer (BC, circles) and renal pelvis cancer (squares). **(B)** Molecular and cellular function annotations of BC urinary EV gene profiles. Ingenuity pathway analysis (IPA) determined the top molecular and cellular functions dysregulated in urinary EV from BC (circles) and NRT (squares) compared to HC/DC. Comparison between BC and NRT are also shown (triangles). Each activation/inactivation status is shown by color based on z score of IPA analysis. The functions activated in BC (z score > 2) but not in NRT (z score < 2) were highlighted in grey. **(C)** Volcano plot analysis. To identify bladder cancer biomarker candidates, volcano plot analysis of the differentially expressed genes was conducted. Top 12 candidates were selected from the genes up regulated in BC compared to HC/DC and NRT and their gene names are shown in red. Statistical analysis result is shown in Supplementary Table 1.

Urinary EV was obtained from patients with bladder cancer (BC, N=4), renal pelvis cancer (RPC, N=2), no residual tumor after transurethral resection of bladder tumor (TURBT) (NRT, N=2), and healthy (HC, N=3) and disease controls (DC, N=1) and applied to RNA-seq analysis (Table 1). Unsupervised clustering analysis of the RNA-seq gene expression data detected several possible clusters corresponding to HC/DC, NRT, BC and RPC (Figure 1A), suggesting that urinary EV mRNA profiles could be used to detect and distinguish BC from non-BC samples. Interestingly, the gene expression profiles of NRT are similar to those of BC and RPC and distinct from those of HC/DC, therefore differential gene expression analysis was conducted in a pairwise manner among BC, NRT and HC/DC. Pathway analysis of the differentially expressed genes suggested cancer- and immune system-related molecular and cellular functions were activated in BC compared to HC/DC (Figure 1B). On the other hand, in NRT, only activation of immune system-related functions was observed and cancer-related functions were not activated (Figure 1B). Volcano plot analysis of the differentially expressed genes identified 12 gene candidates of bladder cancer biomarkers in urinary EV, which were overexpressed in BC compared to HC/DC as well as to NRT: *CEACAM7*, *CRH*, *FABP4*, *GPRC5A*, *HSD17B2*, *KRT17*, *LINC00967*, *OLFM3*, *P4HA1*, *SLC2A1*, *TMEM45A*, and *TMPRSS4* (Figure 1C, Supplementary Table 1).

**Table 1:** Sample information for urinary EV RNA-seq analysis

ID	Disease group	Age	Sex	Stage	Grade	Cytology	Comment
RS01	Healthy control	38	M	-	-	n.d.^a^	
RS02	Healthy control	65	M	-	-	n.d.	
RS03	Healthy control	65	F	-	-	n.d.	
RS04	Disease control	43	M	-	-	n.d.	Atypical epithelium, reddened
RS05	No residual tumor	79	M	-	-	Negative	Previous bladder cancer: pTa, pTis, G1
RS06	No residual tumor	57	F	-	-	Negative	Previous bladder cancer: stage unknown, G1/G2
RS07	Bladder cancer	81	F	pTa	G2	Suspicious	Six recurrences
RS08	Bladder cancer	73	M	pTa	G2	Negative	Five recurrences
RS09	Bladder cancer	81	M	pTa	G2	Negative	First manifestation
RS10	Bladder cancer	64	M	pT4a	G3	Positive	First manifestation, cystectomy
RS11	Renal pelvis cancer	65	F	pT2	G3	Negative	First manifestation, nephrectomy
RS12	Renal pelvis cancer	81	F	pT3	G3	Negative	First manifestation, nephrectomy

Sample information used for urinary EV RNA-seq analysis is shown. Disease group, cancer stages and grades were determined as described in the Materials and Methods. ^a^not determined.

### Urinary EV RT-qPCR analysis

Bladder cancer marker candidates as well as reference genes (*ACTB*, *GAPDH*, *ALDOB*) were assayed by RT-qPCR in 254 urine samples including 173 bladder cancer patient urine samples (Table 2). To select a reference gene, raw gene expression level or real-time PCR threshold cycle (Ct) value of reference gene candidates was analyzed by analysis of variance (ANOVA) (Supplementary Table 3). ANOVA indicated that *ACTB* and *GAPDH* were differentially expressed among the diagnostic groups such as bladder cancer stage and grade. On the other hand, *ALDOB* was highly expressed in urinary EV (mean Ct = 24.1 and median Ct = 23.9) and not differentially expressed among any diagnostic groups such as cancer type, bladder cancer stage and grade, therefore *ALDOB* was selected as a reference gene.

**Table 2:** Sample information for urinary EV mRNA RT-qPCR analysis

	Healthy control (HC)	Disease control (DC)^a^	No residual tumor (NRT)	Bladder cancer (BC)	Renal pelvis cancer (RPC)	Ureter cancer (URC)	Other cancer (OT)^b^
Urine (total 254)	9	9	27	173	26	7	3
Subject (total 208)	9	9	26	131	25	7	3
Age	-						
(mean ± sd)		72.4 ± 10.4	69.3 ± 10.7	72.8 ± 10.6	67.3 ± 12.9	74.1 ± 10.0	76.7 ± 9.3
Gender	-						
Male		7 (78%)	24 (89%)	134 (78%)	21 (81%)	5 (71%)	2 (67%)
Female		2 (22%)	3 (11%)	39 (23%)	5 (19%)	2 (29%)	1 (33%)
Stage	-	-	-				
pTa				115 (67%)	9 (35%)	1 (14.3%)	0 (0%)
pTis				10 (6%)	1 (4%)	0 (0%)	0 (0%)
pT1				37 (21%)	6 (23%)	1 (14.3%)	0 (0%)
> pT2				11 (6%)	10 (39%)	4 (57.1%)	2 (67%)
Not available				0 (0%)	0 (0%)	1 (14.3%)	1 (33%)
Grade	-	-	-				
G1				4 (2%)	0 (0%)	0 (0%)	0 (0%)
G2				110 (64%)	11 (42%)	3 (43%)	0 (0%)
G3				59 (34%)	15 (58%)	4 (57%)	1 (33%)
Not available				0 (0%)	0 (0%)	0 (0%)	2 (67%)
Past cancer history	-						
0		9 (100%)	-	96 (56%)	25 (96%)	6 (86%)	2 (67%)
1		0 (0%)	22 (82%)	40 (23%)	1 (4%)	1 (14%)	1 (33%)
2		0 (0%)	4 (15%)	16 (9%)	0 (0%)	0 (0%)	0 (0%)
> 2		0 (0%)	1 (4%)	21 (12%)	0 (0%)	0 (0%)	0 (0%)
Urine cytology	-						
Positive		1 (11%)	3 (11%)	42 (24%)	4 (15%)	1 (14%)	0 (0%)
Suspicious		2 (22%)	1 (4%)	34 (20%)	12 (46%)	0 (0%)	0 (0%)
Negative		5 (56%)	18 (67%)	81 (47%)	8 (31%)	6 (86%)	3 (100%)
Not available		1 (11%)	5 (19%)	16 (9%)	2 (8%)	0 (0%)	0 (0%)
BTA^c^ [ng/mL] (mean ± sd)	3.0 ± 0.0	7.9 ± 14.8	4.0 ± 2.8	10.5 ± 19.1	15.0 ± 27.6	3.9 ± 2.4	3.0 ± 0.0

Sample information used for urinary EV RT-qPCR analysis is shown. Disease group, cancer stages and grades were determined as described in the Materials and Methods. ^a^DC includes non-cancer patients such as no neoplastic change (N=4), benign epithelium (N=1), chronic pyelonephritis (N=1), inverted papiloma (N=1), MRSA infection (N=1), and inflammatory polyp (N=1). ^b^OT includes non-urothelial cancer patients such as adenocarcinoma (N=1, pT3), renal cell carcinoma (N=1, pT3a, G3), and prostate cancer (N=1). ^c^Bladder Tumor Antigen (BTA) was assayed by BTA ELISA kit (Biotang, MA) following the manufacturer’s protocol.

**Figure 2:**
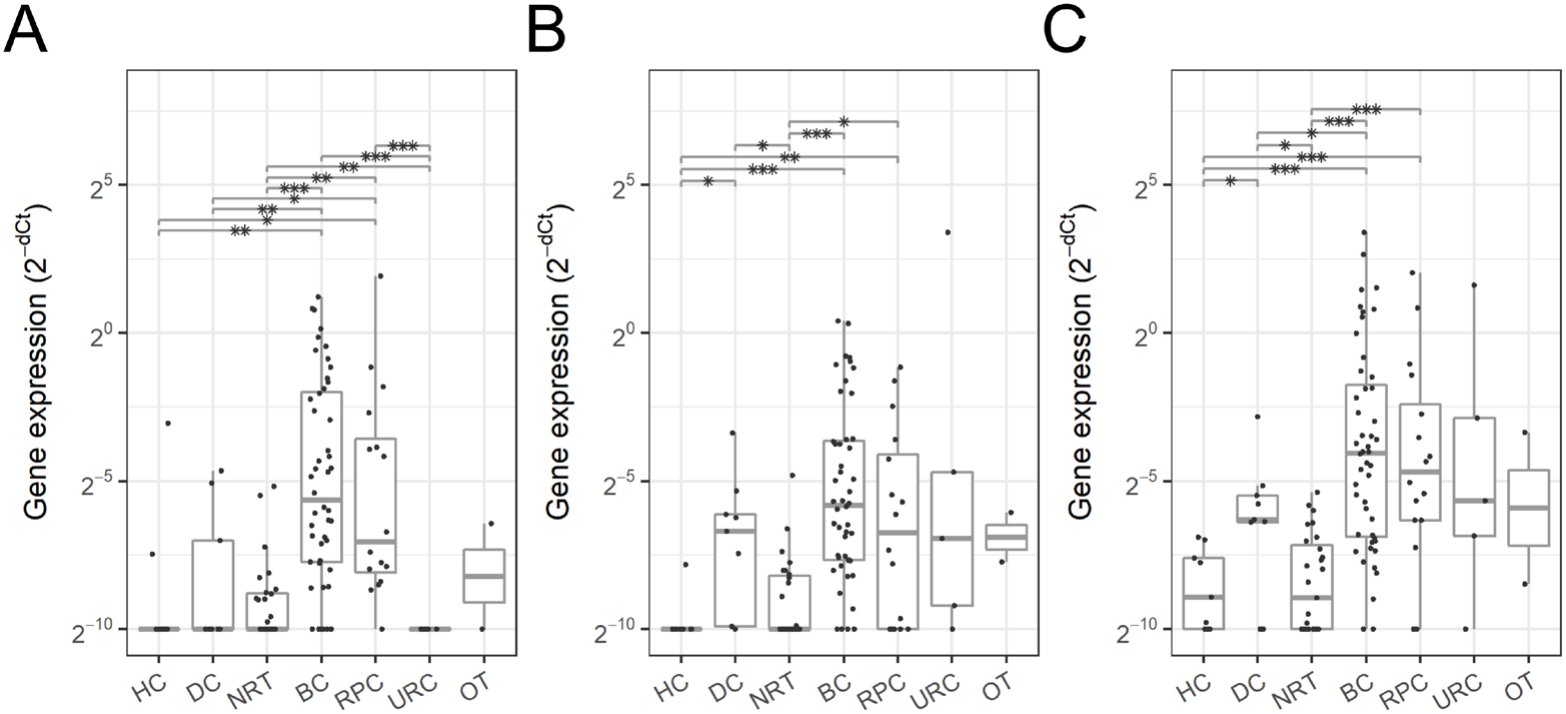
Urinary EV mRNA expression in various cancer types Expression level of *SLC2A1* **(A)**, *GPRC5A* **(B)** and *KRT17* **(C)** in urinary EV was quantified as shown in Materials and Methods, and compared by pT1 and higher stage cancer types shown in Table 2: healthy control (HC), disease control (DC), no residual tumor (NRT), bladder cancer (BC), renal pelvis cancer (RPC), ureter cancer (URC) and non-urothelial cancer (OT). Dots represent individual urine samples. Boxes indicate the first and third quartiles and the horizontal bar in each box represents median and the vertical lines represent minimum and maximum within 1.5 IQRs. Statistical significance was determined by Welchʼs t-test: p value < 0.05 (^*^), < 0.005 (^**^) and < 0.0005 (^***^).

**Figure 3:**
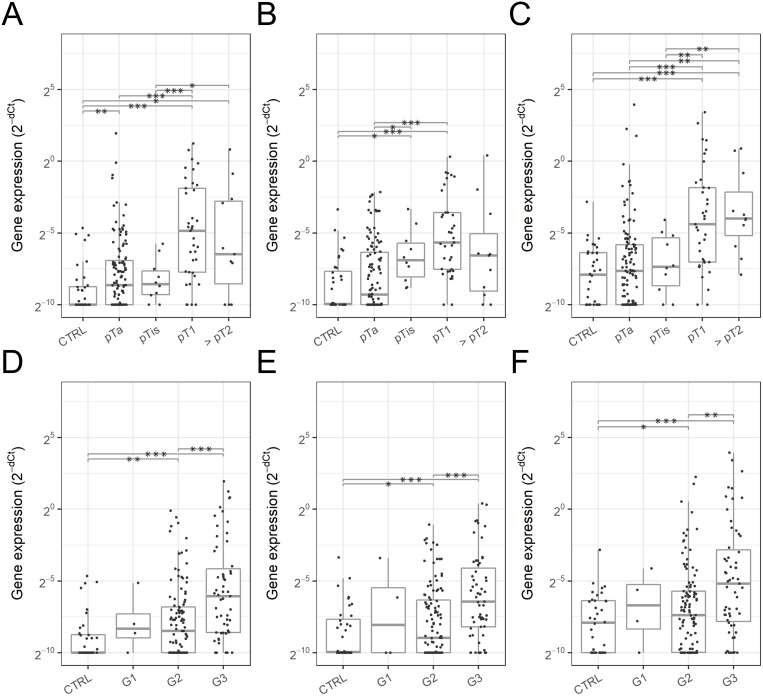
Urinary EV mRNA expression in various stages and grades of bladder cancer Expression level of *SLC2A1* **(A, D)**, *GPRC5A* **(B, E)** and *KRT17* **(C, F)** in urinary EV was compared among the bladder cancer stages (A – C) and grades (D – F) compared to the control groups (DC and NRT). Dots represent individual urine samples. Boxes indicate the first and third quartiles and the horizontal bar in each box represents median and the vertical lines represent minimum and maximum within 1.5 IQRs. Statistical significance was determined by Welchʼs t-test: p value < 0.05 (^*^), < 0.005 (^**^) and < 0.0005 (^***^).

The normalized gene expression profiles were analyzed by disease status such as cancer type (Figure 2, Supplementary Figure 1A), bladder cancer stage (Figure 3A – 3C, Supplementary Figure 1B) and grade (Figure 3D – 3F, Supplementary Figure 1C). Although the other genes tested in this study were identified to be differentially expressed, *SLC2A1*, *GPRC5A* and *KRT17* indicated the most promising results and were selected for further analysis. These three genes were highly expressed in pT1 and higher stage urothelial cancers such as bladder and renal pelvis cancers compared to the non-BC control groups such as HC and NRT (Figure 2). *SLC2A1* expression was elevated by 20.6-fold in pT1 and higher BC and by 7.7-fold in RPC compared to the non-BC control groups and other cancers, indicating that the overexpression of *SLC2A1* is relatively specific to urothelial cancers. *GPRC5A* and *KRT17* were overexpressed in pT1 and higher BC by 18.2-fold and 29.5-fold and in RPC by 9.6-fold and 18.8-fold compared to HC and NRT, however their expression was also elevated in DC including benign tumors and other type of cancers, therefore may be less specific to urothelial cancers but more general to tumors.

The expression level of these three genes was analyzed by bladder cancer stage and grade (Figure 3). *SLC2A1* was significantly overexpressed in pTa (2.6-fold, p = 0.0020), pT1 (35.6-fold, p = 2.0 x 10^-8^), pT2 and higher stages (11.4-fold, p = 0.016), G2 (2.9-fold, p = 0.0018) and G3 bladder cancer (15.2-fold, p = 1.1 x 10^-8^) compared to the non-BC control groups (DC and NRT) (Figure 3A, 3D). *GPRC5A* expression was also significantly elevated in pTis (8.3-fold, p = 0.0084), pT1 (19.5-fold, p = 2.1 x 10^-8^), G2 (2.0-fold, p = 0.048) and G3 bladder cancer (11.5-fold, p = 8.1 x 10^-7^) therefore may be useful to supplement *SLC2A1* especially for the detection of pTis bladder cancer (Figure 3B, 3E). *KRT17* expression was significantly high in pT1 (11.5-fold, p = 2.7 x 10^-7^), pT2 and higher stages (15.0-fold, p = 4.0 x 10^-4^), G2 (1.4-fold, p = 0.022) and G3 bladder cancer (6.7-fold, p = 1.8 x 10^-6^), which was consistent with the expression of *SLC2A1* and *GPRC5A* (Figure 3C, 3F).

### Diagnostic performance analysis of the marker candidates

**Figure 4:**
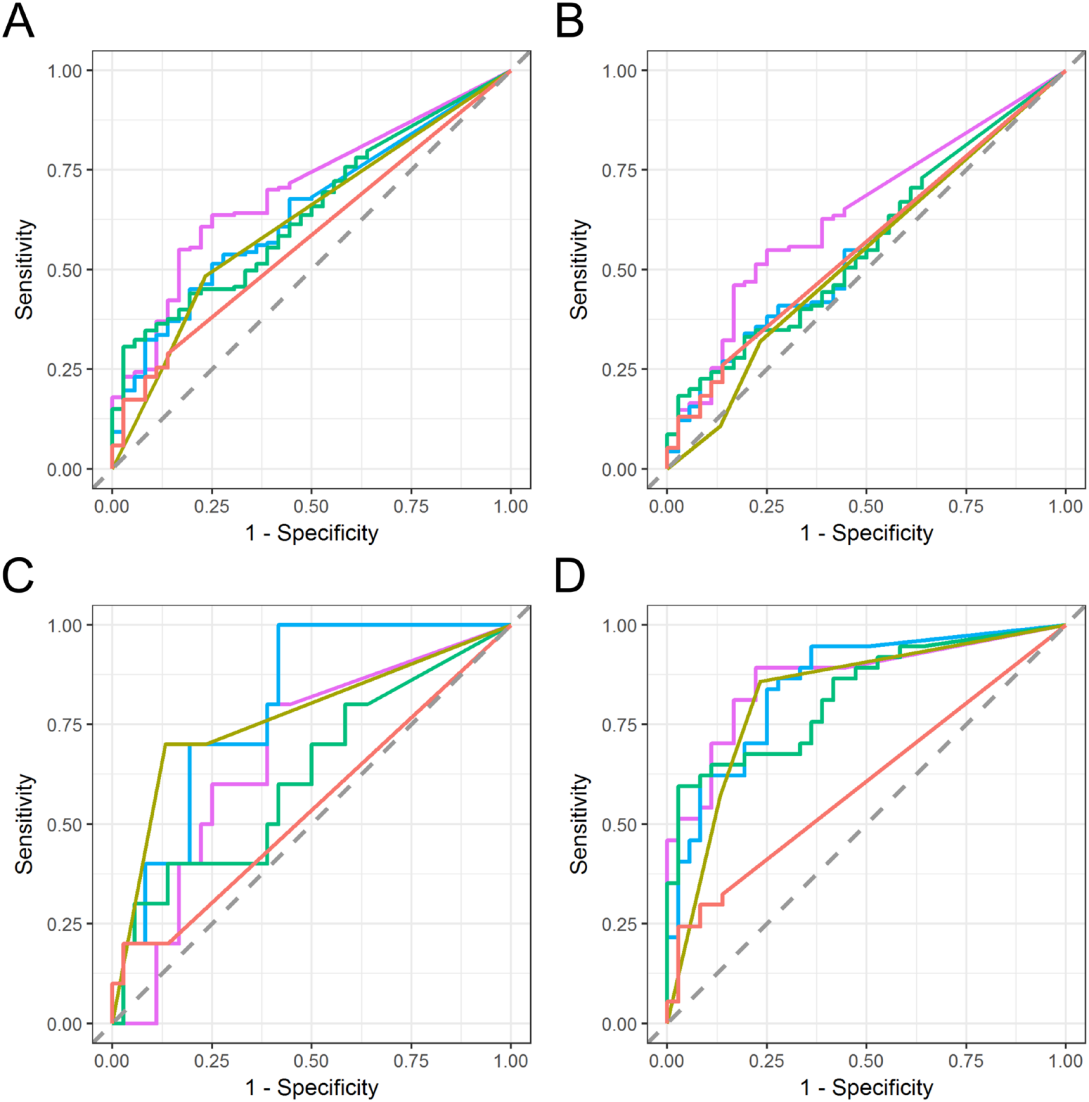
ROC curve analysis of urinary EV mRNA markers in various stages of bladder cancer Diagnostic performance of urinary EV *SLC2A1* (purple), *GPRC5A* (blue) and *KRT17* (green) was evaluated against that of urine cytology (ocher) and BTA ELISA assay (red) for the detection of bladder cancer at various stages. **(A)** all stage bladder cancer (pTa, pTis, pT1 and > pT2), **(B)** pTa bladder cancer, **(C)** pTis bladder cancer, and **(D)** pT1 bladder cancer. Area under the curve (AUC) of ROC curve are shown in Table 3.

The diagnostic performance of these urinary EV mRNA for the detection of bladder cancer was evaluated using ROC curve analysis by comparing various stages/grades of bladder cancer to the control groups (DC and NRT) (Figure 4). Urinary EV mRNA were able to detect both non-muscle invasive bladder cancer (Area under the curve (AUC) 0.64 to 0.70 for all the stages, 0.56 to 0.64 for pTa, 0.62 to 0.80 for pTis, and 0.82 to 0.86 for pT1) and muscle invasive bladder cancer (AUC 0.72 to 0.90 for > pT2) (Table 3). Also, those markers perform better for the detection of G3 bladder cancer (AUC 0.75 to 0.82) than G2 (AUC 0.58 to 0.63) (Table 3). *SLC2A1* outperformed urine cytology and bladder tumor antigen (BTA) especially for the detection of all the stages (AUC 0.70 vs. 0.57 to 0.63), pTa (AUC 0.64 vs. 0.49 to 0.56) and pT1 bladder cancer (AUC 0.86 vs. 0.60 to 0.82). *GPRC5A* also outperformed these conventional bladder cancer markers for the detection of pTis (AUC 0.80 vs. 0.54 to 0.78) and *KRT17* for the detection of pT2 and higher stage bladder cancer (AUC 0.90 vs. 0.66 to 0.72).

**Table 3:** Diagnostic performance of urinary EV mRNA for bladder cancer detection

Stage	Marker	AUC	Sensitivity	Specificity
All (N=173)	*SLC2A1*	0.70 (0.62-0.78)	0.64	0.75
	*GPRC5A*	0.64 (0.56-0.73)	0.54	0.72
	*KRT17*	0.64 (0.56-0.73)	0.58	0.58
	Cytology1	0.62 (0.53-0.71)	0.48	0.77
	Cytology2	0.63 (0.54-0.71)	0.48	0.77
	Cytology3	0.57 (0.50-0.64)	0.27	0.87
	BTA	0.58 (0.52-0.64)	0.29	0.86
pTa (N=115)	*SLC2A1*	0.64 (0.55-0.74)	0.55	0.75
	*GPRC5A*	0.56 (0.46-0.65)	0.55	0.56
	*KRT17*	0.57 (0.46-0.67)	0.63	0.44
	Cytology1	0.53 (0.44-0.63)	0.32	0.77
	Cytology2	0.54 (0.45-0.63)	0.32	0.77
	Cytology3	0.49 (0.42-0.56)	0.11	0.87
	BTA	0.56 (0.49-0.63)	0.26	0.86
pTis (N=10)	*SLC2A1*	0.68 (0.50-0.85)	0.80	0.61
	*GPRC5A*	0.80 (0.67-0.93)	0.70	0.81
	*KRT17*	0.62 (0.41-0.83)	0.60	0.58
	Cytology1	0.77 (0.59-0.94)	0.70	0.87
	Cytology2	0.73 (0.57-0.90)	0.70	0.77
	Cytology3	0.78 (0.62-0.95)	0.70	0.87
	BTA	0.54 (0.39-0.69)	0.20	0.97
pT1 (N=37)	*SLC2A1*	0.86 (0.78-0.95)	0.89	0.78
	*GPRC5A*	0.85 (0.76-0.94)	0.84	0.75
	*KRT17*	0.82 (0.73-0.92)	0.65	0.89
	Cytology1	0.82 (0.72-0.92)	0.86	0.77
	Cytology2	0.81 (0.72-0.91)	0.86	0.77
	Cytology3	0.72 (0.62-0.82)	0.57	0.87
	BTA	0.60 (0.51-0.70)	0.32	0.86
> pT2 (N=11)	*SLC2A1*	0.76 (0.57-0.95)	0.73	0.86
	*GPRC5A*	0.72 (0.54-0.90)	0.64	0.75
	*KRT17*	0.90 (0.79-1.00)	0.82	0.83
	Cytology1	0.72 (0.54-0.91)	0.67	0.77
	Cytology2	0.72 (0.54-0.90)	0.67	0.77
	Cytology3	0.66 (0.47-0.84)	0.44	0.87
	BTA	0.72 (0.55-0.89)	0.55	0.92
G2 (N=110)	*SLC2A1*	0.63 (0.54-0.73)	0.54	0.75
	*GPRC5A*	0.58 (0.48-0.68)	0.58	0.56
	*KRT17*	0.59 (0.49-0.69)	0.55	0.56
	Cytology1	0.52 (0.42-0.61)	0.29	0.77
	Cytology2	0.53 (0.44-0.62)	0.29	0.77
	Cytology3	0.47 (0.41-0.54)	0.08	0.87
	BTA	0.58 (0.51-0.65)	0.28	0.86
G3 (N=59)	*SLC2A1*	0.82 (0.73-0.90)	0.81	0.75
	*GPRC5A*	0.77 (0.68-0.87)	0.71	0.75
	*KRT17*	0.75 (0.65-0.84)	0.56	0.86
	Cytology1	0.81 (0.72-0.90)	0.82	0.77
	Cytology2	0.79 (0.70-0.89)	0.82	0.77
	Cytology3	0.74 (0.65-0.83)	0.61	0.87
	BTA	0.59 (0.51-0.67)	0.31	0.86

Diagnostic performance of urinary EV mRNA, urine cytology and BTA assay for the detection of bladder cancer was evaluated by area under the curve (AUC) with 95% confidence interval in ROC curve analysis. For the optimal threshold, sensitivity and specificity were obtained. DC and NRT (N=36) were used as a control group and BC at various stages/grades was used for a target group. For urine cytology, three different score assignments were used: Cytology1; Positive (2), suspicious (1) and negative (0), Cytology2; Positive/suspicious (1) and negative (0), and Cytology3; Positive (1) and suspicious/negative (0).

To further explore the value of these new markers, the diagnostic performance of these urinary EV mRNA was evaluated under various hypothetical settings such as cytology-negative/suspicious bladder cancer detection (Supplementary Figure 2A), recurrent bladder cancer detection (Supplementary Figure 2B) and non-bladder urothelial cancer detection (Supplementary Figure 2C). In all the cases, the diagnostic performance of urinary EV mRNA outperformed that of conventional urine cytology and BTA assay. *SLC2A1* was able to detect cytology-negative/suspicious bladder cancer (N=115) with AUC 0.68 (Supplementary Figure 2A, Supplementary Table 4) and recurrent bladder cancer (N=77) with AUC 0.62 (Supplementary Figure 2B, Supplementary Table 5). On the other hand, urine cytology and BTA assays were able to detect bladder cancer under the same settings only with AUC 0.50 to 0.59 and AUC 0.51 to 0.53, respectively. For the detection of non-bladder urothelial cancer (RPC and URC, N=32), *KRT17* showed the best diagnostic performance with AUC 0.77 compared to 0.51 to 0.64 for urine cytology and BTA assays (Supplementary Figure 2, Supplementary Table 6).

Sparse logistic regression analysis was employed to further improve the diagnostic performance in combination with urine cytology. These three genes were selected most frequently in the feature selection as well as cytology score (Supplementary Figure 3A, 3B), and a panel of the three genes in combination with urine cytology score was able to detect pT1 and higher stage bladder cancer with AUC 0.93 ± 0.04 as well as other stages and settings (Supplementary Figure 3C, 3D).

## DISCUSSION

In this study, RNA-seq analysis of urinary EV from bladder cancer patients was performed and indicated cancer- and immune system-related functions were activated in BC urinary EV. Interestingly immune system-related functions were activated in NRT urine although urine was collected more than four months after BCG intravesical treatment or without any immune related treatment. This may suggest that their bladder environments may be exposed to carcinogenic compounds constantly and cause high recurrence of cancer however further studies are necessary to exclude other possibilities. From the RNA-seq data, the top 12 bladder cancer marker candidates were identified and further analyzed in a separate cohort of 254 urine samples by RT-qPCR. We selected three promising urinary EV markers, *SLC2A1*, *GPRC5A* and *KRT17*, and confirmed that the diagnostic performance of these genes outperformed those of conventional urine cytology and BTA assays. These genes may serve as biomarkers not only for bladder cancer but also for other urothelial cancers such as renal pelvis and ureter cancers. To investigate the practical values of these genes, the diagnostic performance of these genes was also evaluated under hypothetical clinical settings, suggesting these genes may be supplementary to urine cytology as these genes can detect bladder cancer even in the patient population whose cytology results are negative or suspicious. Indeed, the combination of the three genes and urine cytology were the most selected features in logistic regression analysis and improved the diagnostic performance further for pT1 and higher bladder cancer. Urinary EV markers are also supplementary to urine cytology in terms of assay procedure because EV mRNA are assayed using urinary supernatant that can be obtained following urine cell/cast precipitation for urine cytology. Therefore, these urinary EV mRNA may be a practical option to monitor recurrence after TURBT non-invasively in combination with urine cytology. It is still challenging to detect pTa bladder cancer (*SLC2A1*, AUC 0.64 (95% CI 0.55 – 0.74)) since 87% of the pTa samples were lower grades (G1/G2) although those cancers have a low risk for recurrence, while high-risk pTa/G3 bladder cancer may be detected with better diagnostic performance (*SLC2A1*, AUC 0.76 (95% CI 0.62 – 0.91)).

These three genes and corresponding proteins have been studied well in several types of cancers. *SLC2A1* (solute carrier family 2 member 1) encodes glucose transporters 1 (Glut1). Glut1 is overexpressed in many types of cancers including urothelial cancers and involved with glucose uptake to support the growth and proliferation of cancer cells [13]. In a large scale meta-analysis of 26 studies (2948 patients), overexpression of Glut1 in solid tumors significantly correlates with poor 3- and 5-year overall survivals [14]. Therefore, urinary EV *SLC2A1* expression may be associated with the prognosis of urothelial cancer patients. *GPRC5A* (G protein-coupled receptor class C group 5 member A) encodes retinoic acid-induced protein 3 (RAI3), which is associated with many types of cancers [15]. RAI3 behaves as a tumor suppressor and its repression is associated with poor lung cancer prognosis [16]. On the other hand, overexpression of RAI3 in colon [17], liver [18], gastric [19] and pancreatic cancers [20] is associated with poor prognosis. *KRT17* (keratin 17) has been reported to be overexpressed not only in bladder cancer [21] but also in other types of cancers such as breast [22], cervical [23], oral [24], esophagus [25], pancreatic [26] and colon cancers [27], and associated with poor prognosis of breast [22] and cervical cancers [23]. In oral cancer, *KRT17* stimulates the Akt/mTOR pathway and upregulates *SLC2A1* and glucose uptake which facilitates tumor growth [24]. These studies support the rationality for the overexpression of EV *SLC2A1*, *GPRC5A* and *KRT17* in bladder cancer urine, however further study is necessary to determine if these EV mRNA are originated from tumors or associated with the overexpression of corresponding proteins in tumors.

Recent multiomics studies reported that ‘basal’ and ‘luminal’ subtypes of muscle invasive bladder tumors showed distinct tumor gene expression profiles and ‘basal’ subtype showed more progressive and worse prognosis than the other subtypes [28]. Although the subtype information is not available in this study, the expression levels of *SLC2A1* and *KRT17* in bladder tumors are moderately correlated with the ‘basal’ markers such as *KRT16*, *KRT6A*, *KRT6C*, *KRT5*, *CDH3*, *KRT6B*, and *KRT14*, while the expression level of *GPRC5A* is weakly correlated with the ‘luminal’ markers such as *KRT19* and *ERBB3* (Supplementary Figure 4, Supplementary Table 7), therefore it may be possible to identify progressive ‘basal’ subtype by urinary EV analysis.

There are several previous studies for non-invasive bladder cancer biomarkers. Perez, *et al.* discovered several urinary EV marker candidates such as *LASS2* and *GALNT1* [29] although these genes were not detected or differentially expressed in our RNA-seq data therefore not investigated further. Christensen *et al.* recently reported that *FGFR3* and *PIK3CA* mutation in urinary cell free DNA allows detection of non-muscle invasive bladder cancer including risks of progression and recurrence [30]. Although our study did not analyze cell free DNA due to the sample availability, it may be useful to analyze other urinary markers to improve the diagnostic performance further.

The present feasibility study has several limitations. First, the patient population is skewed especially the number of no remained tumor control was very low compared to that of bladder cancer patients because the patients at our facility were highly suspected to have urothelial cancer based on the previous test results. Consequently, we were not able to investigate the possibility of replacing cystoscopy with these markers. Second, we were not able to obtain sufficient numbers of samples for some of the diagnostic categories especially for pTis and pT2 and higher bladder cancer due to the time constraint of sample collection and study design. To overcome aforementioned limitations, a 3-year prospective multi-center study is ongoing to validate this preliminary study result especially for bladder cancer recurrence after TURBT.

In this study, three urinary EV mRNA were discovered to be elevated in bladder cancer and used to determine diagnostic performance in non-muscle and muscle invasive bladder cancer. Compared to the conventional urine cytology and BTA assays, these three markers showed promising diagnostic performances for the detection of bladder cancer especially at earlier stages. This feasibility study suggested that urinary EV mRNA are promising biomarkers of urothelial cancer and worth further investigation.

## MATERIALS AND METHODS

### Patient and sample recruitment

This study was reviewed and approved by the institutional review board at Sapporo City General Hospital (approval no. H25-047-197). Patients eligible for the study were suspected urothelial cancer patients based on the presence of hematuria, irritable bladder symptoms, or the test result of cystourethroscopy, computed tomography and/or abdominal echogram. Up to 15 mL spot urine was collected prior to TURBT with informed consent at our facility between May 2014 and May 2016 (Tables 1, 2). We excluded patients whose pathology record was not available. For healthy control, spot urine was collected from healthy donors anonymously. The urine samples were stored at -80°C within 3 hours after the collection.

Urothelial cancer was diagnosed by cystoscopy, urine cytology and pathological diagnosis of tumors obtained by means of transurethral resection or radical excision. Cancer staging was determined by the World Health Organization 2004 criteria and grading was done by the World Health Organization 1999 criteria. No residual tumor was defined as no sign of bladder cancer after the last transurethral resection of tumors. Non-cancer disease control includes the patients who was originally suspected for urothelial cancer but confirmed absence of cancer. Urine cytology specimens were evaluated by a cytopathologist and three experienced cytotechnologists following the Papanicolaou classification system and negative urine cytology was defined to be class I and II, suspicious cytology was to be class III and IV and positive cytology was to be class V.

### Urine EV RNA-seq analysis

Urine samples were obtained from 12 patients and donors (Table 1). Urinary EV was isolated by the exosome isolation tube (Hitachi Chemical Diagnostics, Inc., CA) as previously described [10]. The captured EV were lysed on the filter tip, and the resultant lysates were transferred by centrifugation to a T7 promoter oligo(dT)-immobilized microplate for mRNA hybridization. The hybridized mRNA was amplified by MEGAscript T7 Transcription Kit (Life Technologies, CA) directly on the plate. RNA was purified using RNeasy MinElute Cleanup kit (Qiagen, CA) before being used as starting material for TruSeq library preparation (Illumina, CA). The RNA-seq libraries were sequenced on an Illumina HiSeq 2500 instrument using single read 50 base pair chemistry. After the obtained raw reads were filtered and deduplicated by FASTX-Toolkit and mapped against the human genome (GRCh38) by TopHat, the read counts were obtained by HTSeq and analyzed by edgeR. Additionally, ingenuity pathway analysis (IPA) (Qiagen, CA) was employed to identify dysregulated pathways.

### Urinary EV mRNA assay for RT-qPCR

Urine samples were obtained from urothelial cancer and other patients and healthy donors (N=254) (Table 2). Urinary EV mRNA assay was conducted as previously described except the use of 10 μM random hexamer at cDNA synthesis step [10]. The primer sequences are available in Supplementary Table 2. Threshold cycle (Ct) values of the marker candidates were normalized by that of reference gene (*ALDOB*) using the delta Ct method. Data analysis was performed using R. Statistical significance was obtained by Welch’s t-test with p value < 5%. Diagnostic performance was evaluated and compared to urine cytology and bladder tumor antigen assays by the area under the curve (AUC) in ROC curve analysis. Optimum threshold was obtained at the nearest point of the ROC curve to the top-left corner, and used to calculate sensitivity and specificity to characterize diagnostic performance of marker candidates.

## SUPPLEMENTARY MATERIALS FIGURES AND TABLES



## Abbreviations

(EV): Extracellular vesicles
(TURBT): Transurethral resection of bladder tumor
(AUA): Area under the curve
(BC): Bladder cancer
(RPC): Renal pelvis cancer
(URC): Ureter cancer
(OT): Other cancer
(NRT): No residual tumor
(DC): Disease control
(HC): Healthy control
(BTA): Bladder tumor antigen
(RNA-seq): RNA sequencing
(RT-qPCR): Quantitative reverse transcription polymerase chain reaction
