# Supplementary material for: Bladder cancer detection by urinary extracellular vesicle mRNA analysis
Source: Oncotarget. 2018 Aug 28;9(67):32810–21. doi: 10.18632/oncotarget.25998 (PMC6132352; doi:10.18632/oncotarget.25998)
Supplement: Supplementary file 1 [file oncotarget-09-32810-s001.pdf]

Bladder cancer detection by urinary extracellular vesicle mRNA analysis

SUPPLEMENTARY MATERIALS

A. Cancer type (pT1 and higher stages only)

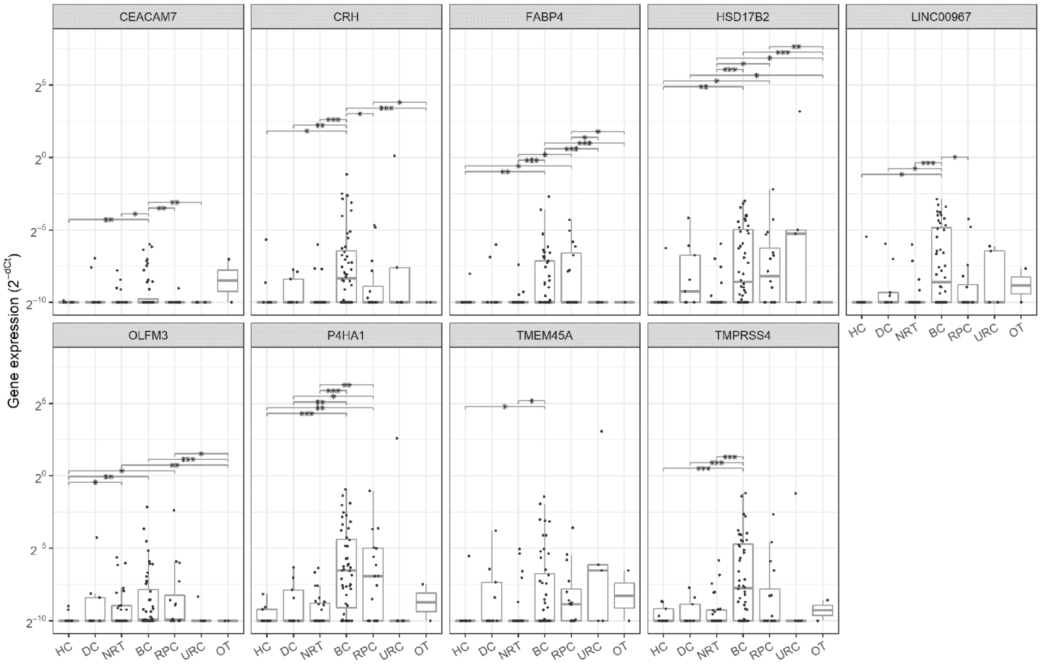

B. Bladder cancer stage

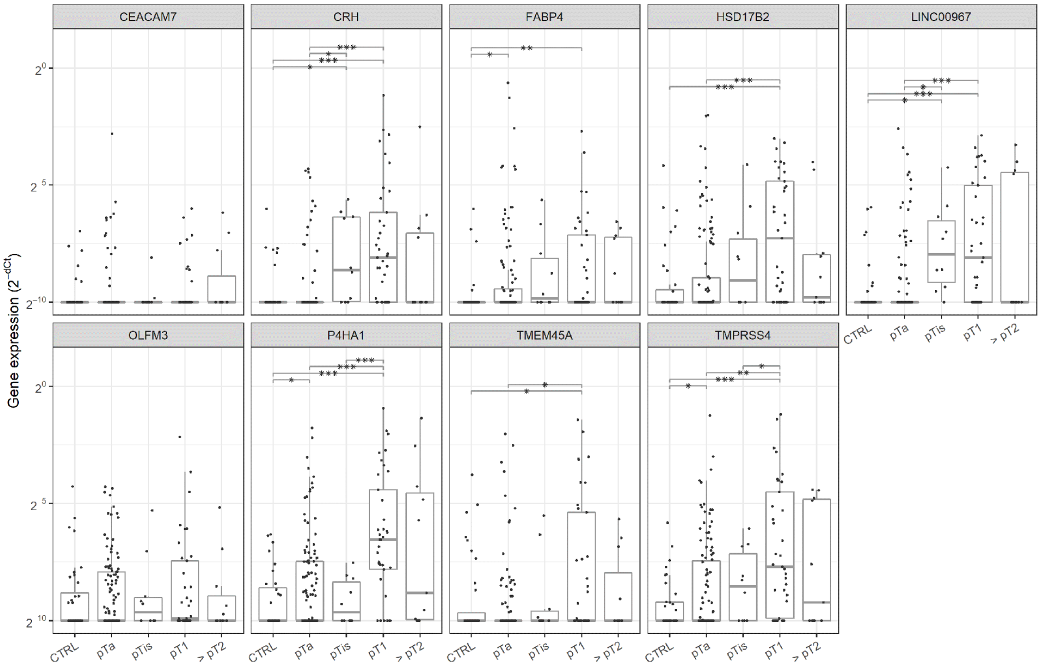

(*Continued*)

C. Bladder cancer grade

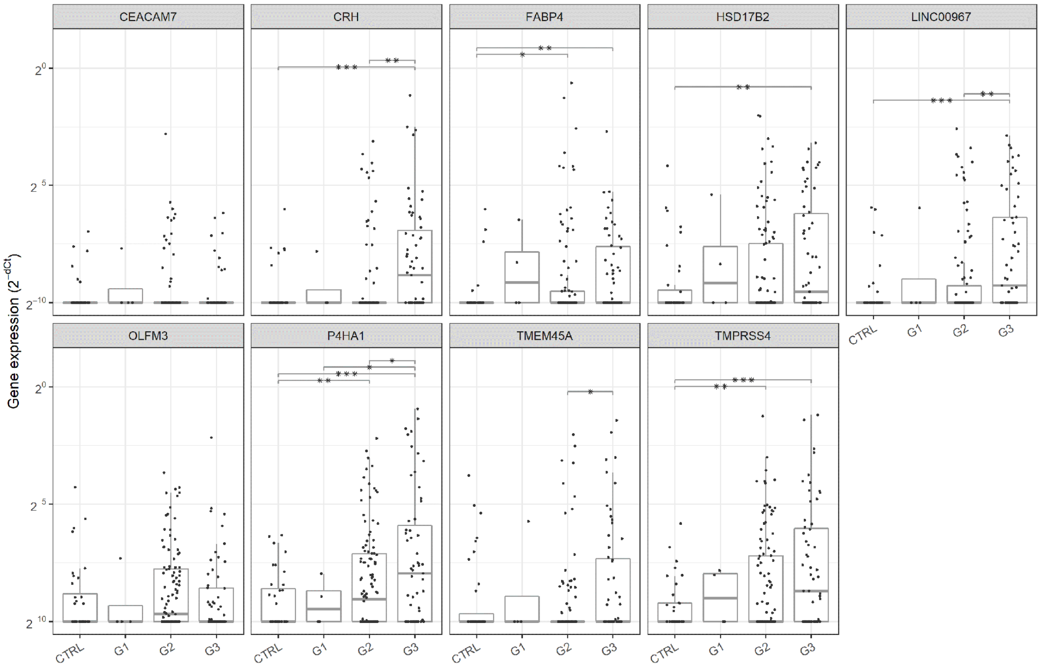

Supplementary Figure 1 (*Continued*): Gene expression of urinary EV mRNA marker candidates. Expression level of the marker candidates in urinary EV from 254 urine samples was analyzed by cancer type (pT1 and higher stages only) **(A)**, bladder cancer stage **(B)** and grade **(C)**. Control groups in B and C are DC and NRT. Dots represent individual urine samples. Boxes indicate the first and third quartiles and the horizontal bar in each box represents median and the vertical lines represent minimum and maximum within 1.5 IQRs. Statistical significance was determined by Welch's t-test. Red and black bars indicate p value < 0.01 and < 0.05, respectively.

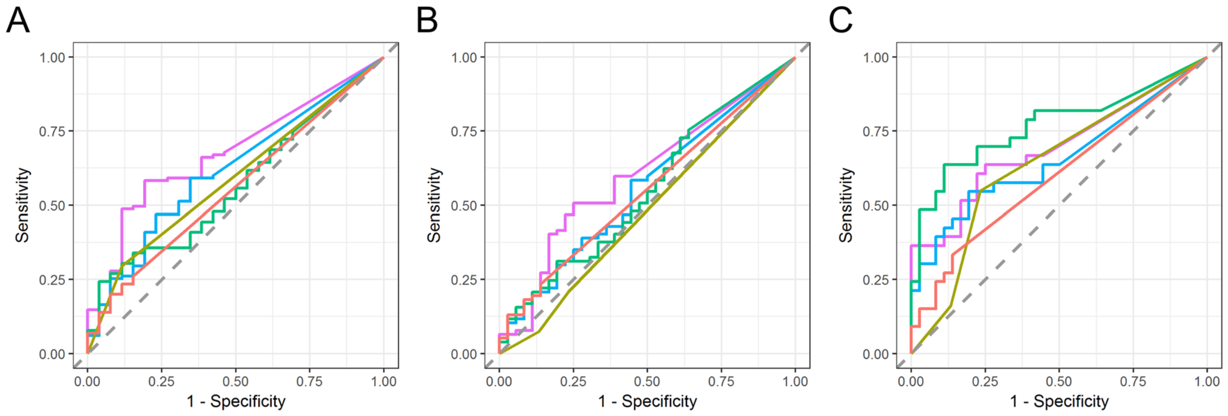

Supplementary Figure 2: ROC curve analysis of urinary EV mRNA markers. Diagnostic performance of urinary EV *SLC2A1* (purple), *GPRC5A* (blue) and *KRT17* (green) was evaluated against those of urine cytology (ocher) and BTA ELISA assay (red) in various settings. **(A)** cytology-negative bladder cancer, **(B)** recurrent bladder cancer and **(C)** renal pelvis and ureter cancer. Area under the curve (AUC) are shown in Supplementary Table 4, Supplementary Table 5 and Supplementary Table 6, respectively.

A. Top features selected in 5000 repeats of SLR

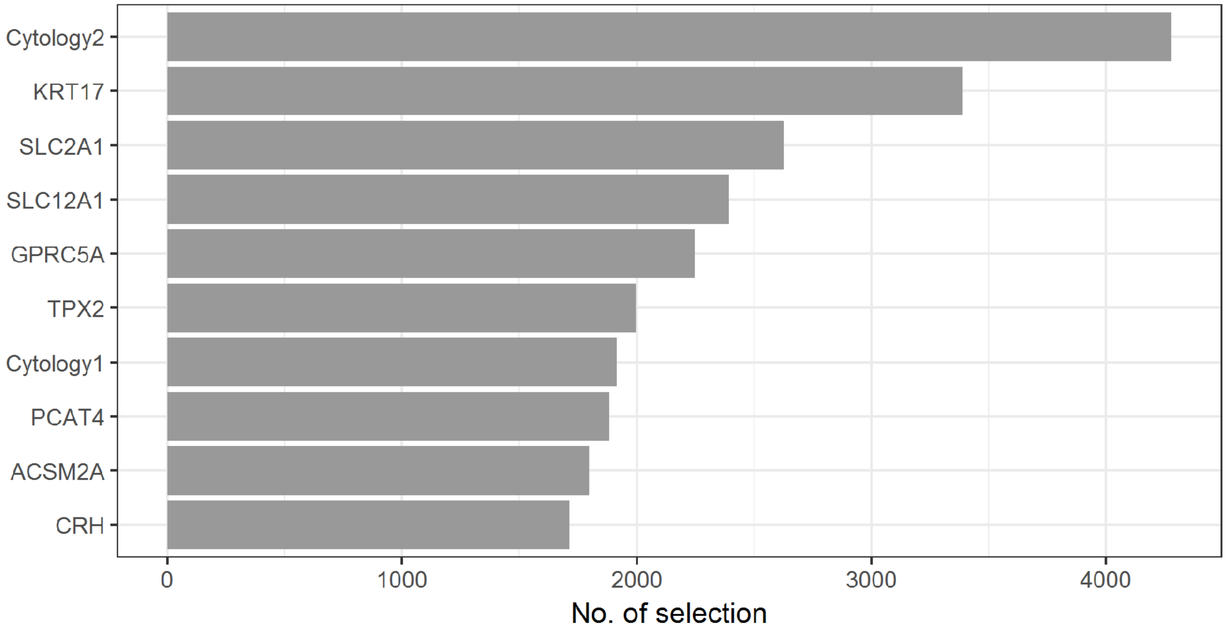

B. Top formulas selected in 5000 repeats of SLR

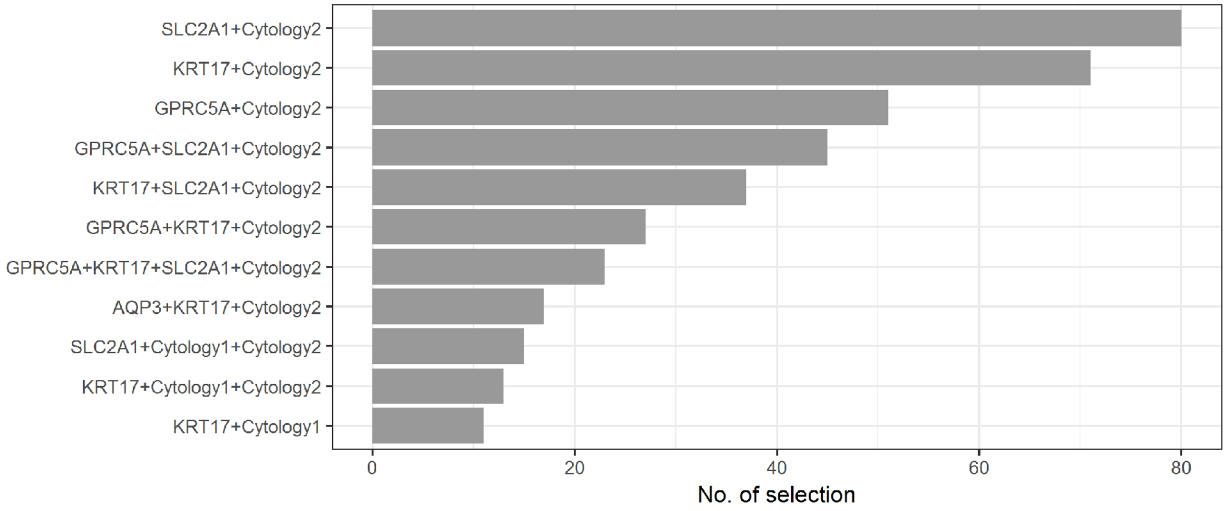

C. Diagnostic performance of top formulas (pT1 and higher stage bladder cancer)

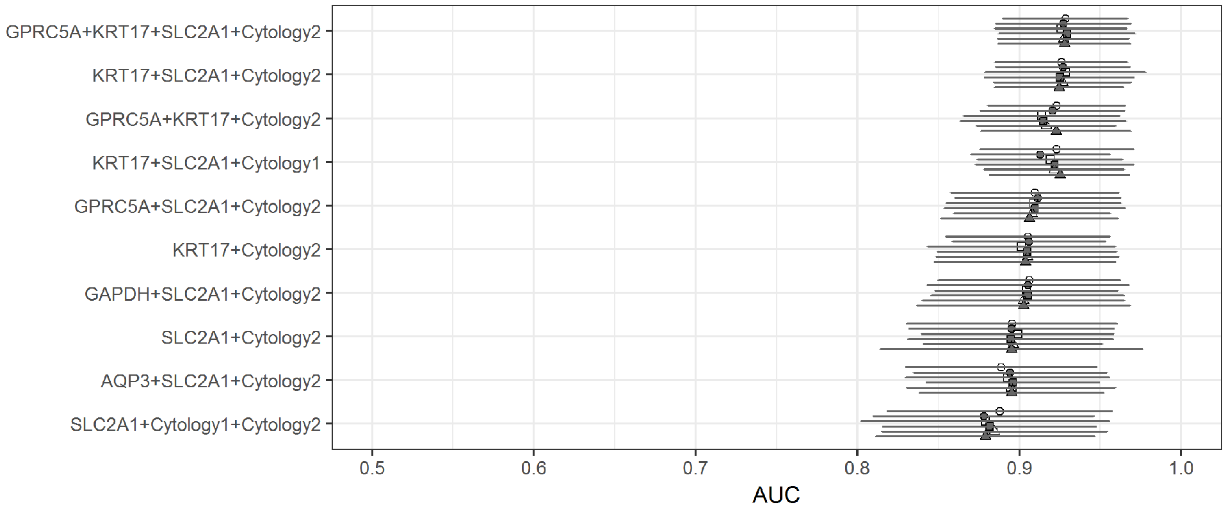

(*Continued*)

D. Diagnostic performance of the selected formula

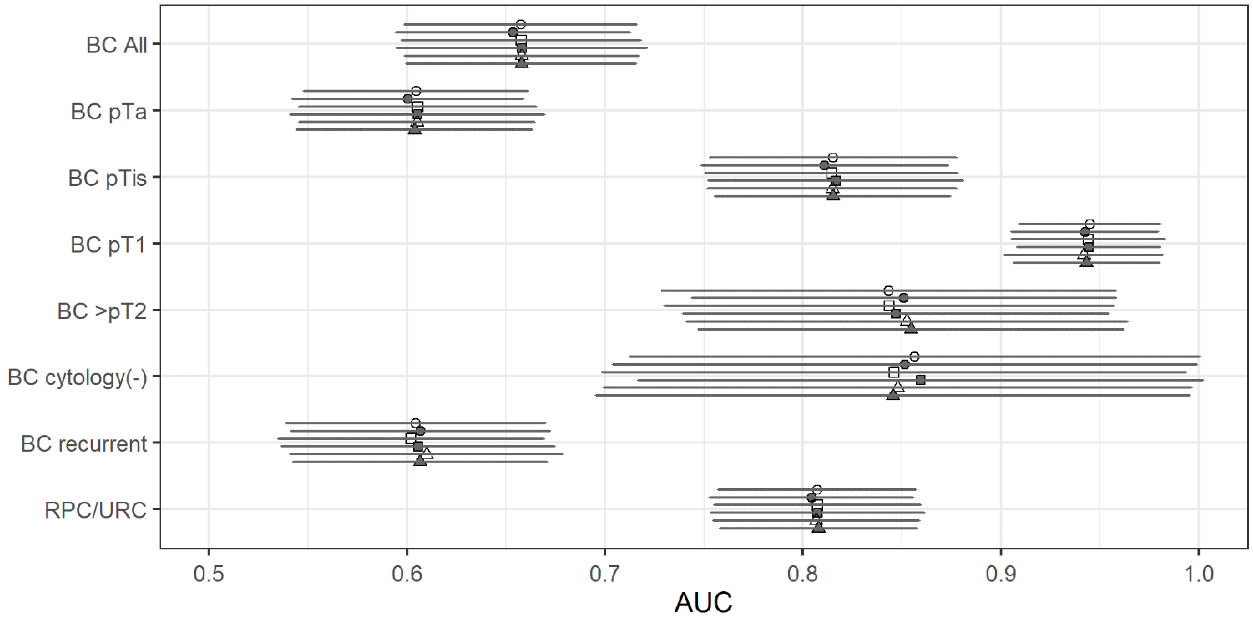

Supplementary Figure 3 (*Continued*): Sparse logistic regression analysis. Sparse logistic regression analysis was conducted by glmnet [32]. *ALDOB* normalized delta Ct values of the 15 genes analyzed in this study, additional 45 genes selected from the RNA-seq data and urine cytology scores were used for feature selection. For urine cytology, three different scorings were used: Cytology1; Positive (2), suspicious (1) and negative (0), Cytology2; Positive/suspicious (1) and negative (0), and Cytology3; Positive (1) and suspicious/negative (0). Logistic regression analysis with feature selection was performed to determine if the sample is bladder cancer positive (score 1) or negative (score 0). For bladder cancer positive, pT1 and higher stage bladder cancer samples were used, and DC and NRT samples were used for bladder cancer negative. Logistic regression analysis was conducted with 10-fold cross validation and 5000 bootstrap re-sampling, and the top features and formulas selected the most were shown in **(A)** and **(B)**, respectively. For each of the top formulas, diagnostic performance of pT1 and higher stage bladder cancer or mean AUC (± s.d.) was obtained through 10-fold cross validation and 500 bootstrap re-sampling with random noise addition with varying s.d. (0: open circle, 0.1: closed circle, 0.25: open square, 0.5: closed square, 0.75: open triangle and 1: closed triangle) for further validation as shown in **(C).** The top formula, ‘GPRC5A+KRT17+SLC2A1+Cytology2”, showed AUC 0.93 ± 0.04 for pT1 and higher bladder cancer detection and improved the diagnostic performance further with various stages and settings as shown in **(D)**.

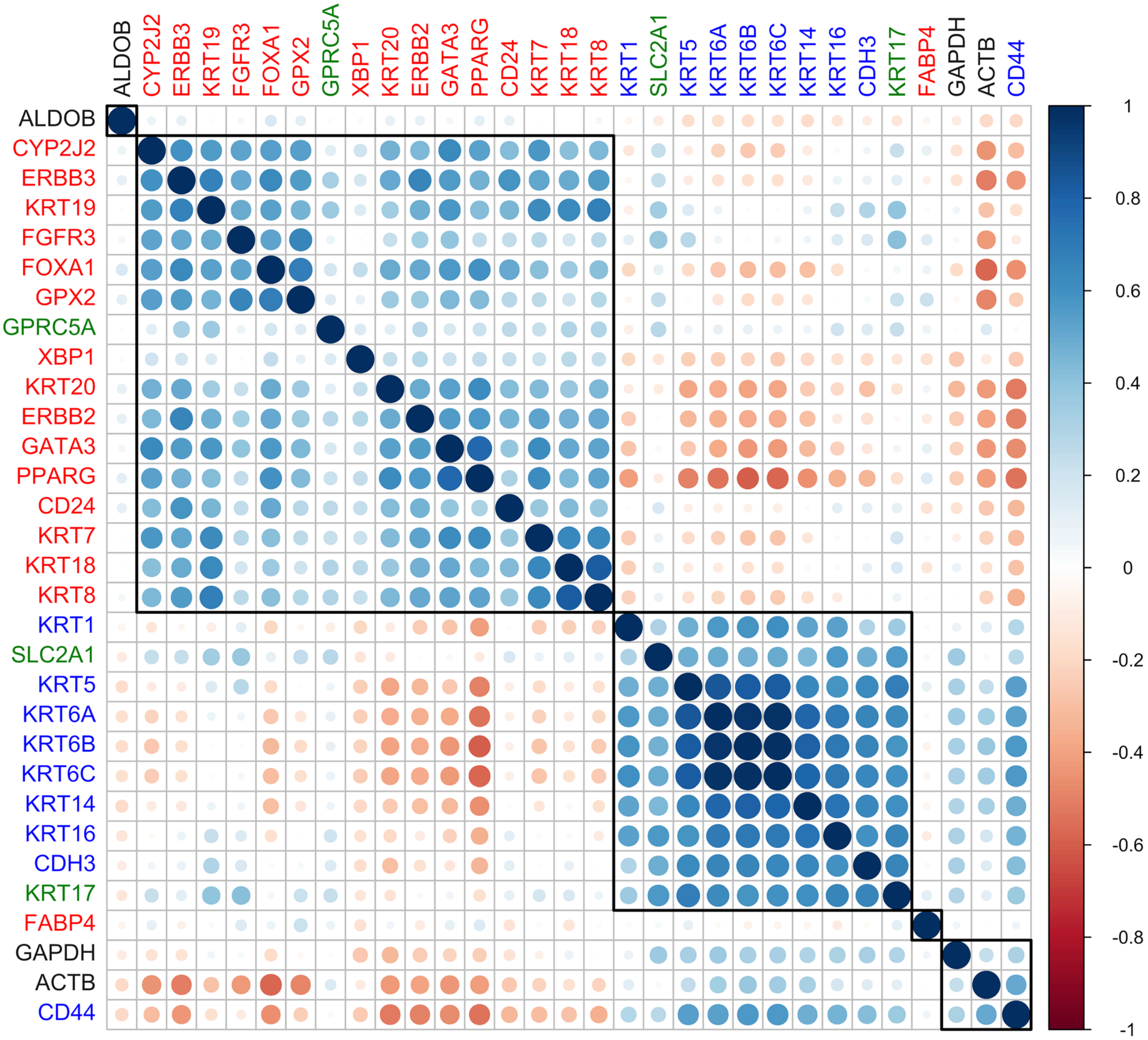

Supplementary Figure 4: Gene expression of urinary EV mRNA marker candidates. Correlation analysis was conducted to investigate the similarity of gene expression in bladder tumors (TCGA [31]) for the markers identified in this study (*SLC2A1*, *GPRC5A* and *KRT17*) (shown in green), reference genes (*ACTB*, *GAPDH*, and *ALDOB*) (shown in black), and 'basal' (shown in blue) and 'luminal' (shown in red) subtype markers Choi *et al.* reported [28]. Pearson correlation coefficients (r) are shown by the colored circles and clusters are shown by rectangles (Supplementary Table 7). The expression level of *SLC2A1* was positively correlated with those of *KRT17*, *KRT16*, *KRT6A*, *KRT6C*, *KRT5*, *CDH3*, *KRT6B*, *KRT14*, *FGFR3*, *GAPDH*, *KRT19*, and *KRT1* ($r > 0.3$ and p value $< 1.0 \times 10^{-10}$). The expression level of *KRT17* was positively correlated with those of *KRT5*, *CDH3*, *KRT16*, *KRT6A*, *KRT14*, *KRT6C*, *KRT6B*, *SLC2A1*, *FGFR3*, *KRT19*, *CD44*, and *KRT1* ($r > 0.3$ and p value $< 1.0 \times 10^{-10}$). The expression level of GPRC5A was correlated with those of *KRT19* and *ERBB3* ($r > 0.3$ and p value $< 1.0 \times 10^{-10}$).

Supplementary Table 1: Differential gene expression analysis of RNA-seq data

Gene	*Bladder Cancer vs. Healthy/Disease Controls*				*Bladder Cancer vs. No Residual Tumor*			
	**logFC**	**logCPM**	**p value**	**FDR**	**logFC**	**logCPM**	**p value**	**FDR**
FABP4	9.27	3.73	3.68E-05	0.0555	4.53	3.73	5.04E-02	1
CRH	8.59	5.67	6.12E-07	0.0081	9.11	5.67	3.63E-04	0.5984
CEACAM7	8.16	3.95	4.72E-05	0.0577	4.23	3.95	5.47E-02	1
LINC00967	7.77	6.28	7.11E-06	0.0235	5.10	6.28	2.12E-02	1
HSD17B2	7.43	4.64	4.62E-04	0.2037	4.12	4.64	9.20E-02	1
OLFM3	6.65	3.75	1.40E-04	0.1058	3.97	3.75	6.95E-02	1
TMEM45A	6.53	6.06	1.28E-04	0.1055	6.78	6.06	5.69E-03	1
KRT17	6.16	5.92	1.15E-06	0.0101	1.32	5.92	3.38E-01	1
GPRC5A	4.63	5.26	3.28E-04	0.1922	2.91	5.26	8.59E-02	1
TMPRSS4	4.61	5.06	4.18E-05	0.0555	3.58	5.06	1.53E-02	1
P4HA1	4.47	7.29	7.47E-06	0.0235	2.19	7.29	7.91E-02	1
SLC2A1	4.20	7.79	4.42E-04	0.2011	4.75	7.79	6.70E-03	1

Differential gene expression analysis of urinary EV was conducted by edgeR and the top 12 bladder cancer marker candidates were selected. The difference of expression between the groups is shown by log2 fold change (logFC), the mean expression levels of the genes is shown in log2 count per million (logCPM) as well as p value and false discovery rate (FDR).

Supplementary Table 2: Primer sequence list

Gene	Sense primer (5' to 3')	Antisense primer (5' to 3')
ACTB	tttttcctggcacccagcacaat	tttttgccgatccacacggagtact
ALDOB	aaccaccattcaagggcttg	ttggcgttttcctggatagc
CEACAM7	tcagcgccacaaagaatgac	aggtcaggtgaacttgcttg
CRH	atctccctggatctcaccttc	tgtgagcttgctgtgctaac
FABP4	cctggtacatgtgcagaaatgg	acgcctttcatgacgcattc
GAPDH	cccactcctccacctttgac	cataccaggaaatgagcttgacaa
GPRC5A	gctcatgcttcctgactttgac	ttgtgagcagccaaaactcg
HSD17B2	tttttaacaatgcatggccgtgaac	tttttatgctgctgacattcaccag
KRT17	tggacaatgccaacatcctg	tcaaacttggtgcggaagtc
LINC00967	tggagatggttggggtcaaatc	tgcatccacaaagcacactg
OLFM3	accaaagagtgctgagcttg	tcatccaagcaccaaatcgg
P4HA1	agttggagctagtgtttggc	ttgttgccaactagcactgg
SLC2A1	tcattgtgggcatgtgcttc	accaggagcacagtgaagatg
TMEM45A	acatctttgtgcaccagctg	aaggaactctaggaaggcaacg
TMPRSS4	agatgatgtgtgcaggcatc	acatgccactggtcagattg

Supplementary Table 3: ANOVA analysis of reference gene expression

Gene	Threshold cycle		p value (ANOVA)		
	**mean**	**median**	**Type**	**Stage**	**Grade**
ACTB	25.6	25.8	n.s.	$7.3x10^{-7}$	$1.3x10^{-3}$
GAPDH	26.0	26.2	n.s.	$2.0x10^{-7}$	$1.6x10^{-4}$
ALDOB	24.1	23.9	n.s.	n.s.	n.s.

Ten reference gene candidates were selected from ubiquitously expressed genes, and kidney specific genes and their raw threshold cycle values in the RT-qPCR assay were analyzed by ANOVA among the different diagnostic groups such as type of cancer (Type), bladder cancer stages (Stage) or bladder cancer grades (Grade). P values above 0.05 were considered not significant (n.s.).

Supplementary Table 4: Diagnostic performance of urinary EV mRNA for bladder cancer detection in urine cytology negative or suspicious population

**Marker**	**All**	**pTa**	**pTis**	**pT1**	**> pT2**
	**N=115**	**N=92**	**N=3**	**N=15**	**N=5**
Cytology1	0.59	0.56	0.56	0.78	0.64
Cytology2	0.59	0.56	0.56	0.78	0.64
Cytology3	0.50	0.50	0.50	0.50	0.50
BTA	0.56	0.56	0.62	0.53	0.63
SLC2A1	0.68	0.64	0.87	0.84	0.67
GPRC5A	0.62	0.57	0.81	0.86	0.65
KRT17	0.57	0.53	0.60	0.74	0.89

Diagnostic performance of urinary EV mRNA for the detection of bladder cancer was evaluated in the patient population whose urine cytology result was not positive. AUC in ROC curve analysis is shown in the table. DC and NRT with suspicious or negative cytology results (N=26) were used as a control group and BC with suspicious or negative cytology results was used for a target group. For urine cytology, three different score assignments were used: Cytology1; Positive (2), suspicious (1) and negative (0), Cytology2; Positive/suspicious (1) and negative (0), and Cytology3; Positive (1) and suspicious/negative (0).

Supplementary Table 5: Diagnostic performance of urinary EV mRNA for recurrent bladder cancer detection

**Marker**	**All**	**pTa**	**pTis**	**pT1**
	**N=77**	**N=67**	**N=5**	**N=10**
Cytology1	0.51	0.52	0.62	0.64
Cytology2	0.51	0.52	0.61	0.66
Cytology3	0.53	0.55	0.63	0.51
BTA	0.55	0.53	0.66	0.60
SLC2A1	0.62	0.56	0.81	0.86
GPRC5A	0.62	0.56	0.84	0.91
KRT17	0.62	0.60	0.60	0.74

Diagnostic performance of urinary EV mRNA for the detection of bladder cancer was evaluated in the patient population who experienced bladder cancer previously. AUC in ROC curve analysis is shown in the table. NRT (N=27) was used as a control group and recurrent BC was used for a target group. For urine cytology, three different score assignments were used: Cytology1; Positive (2), suspicious (1) and negative (0), Cytology2; Positive/suspicious (1) and negative (0), and Cytology3; Positive (1) and suspicious/negative (0). > pT2 (N=0) was excluded from analysis due to the lack of samples.

Supplementary Table 6: Diagnostic performance of urinary EV mRNA for renal pelvis and ureter cancer detection

Marker	All	pTa	pT1	> pT2
	N=32	N=10	N=7	N=14
Cytology1	0.64	0.70	0.79	0.52
Cytology2	0.66	0.72	0.81	0.54
Cytology3	0.51	0.54	0.58	0.57
BTA	0.60	0.58	0.56	0.58
SLC2A1	0.70	0.58	0.77	0.70
GPRC5A	0.66	0.60	0.74	0.67
KRT17	0.77	0.66	0.87	0.78

Diagnostic performance of urinary EV mRNA for the detection of non-bladder urothelial cancer (RPC and URC) was evaluated. AUC in ROC curve analysis is shown in the table. DC and NRT (N=36) was used as a control group and RPC and RPC were used for a target group. For urine cytology, three different score assignments were used: Cytology1; Positive (2), suspicious (1) and negative (0), Cytology2; Positive/suspicious (1) and negative (0), and Cytology3; Positive (1) and suspicious/negative (0). pTis (N=1) was excluded from analysis due to the small number of samples.

Supplementary Table 7: Correlation analysis of markers in bladder tumors

Marker	Gene	Type	Pearson's r	p value
SLC2A1	*KRT17*	Marker	0.57	< 1.0E-17
	KRT16	Basal	0.56	< 1.0E-17
	KRT6A	Basal	0.50	< 1.0E-17
	KRT6C	Basal	0.49	< 1.0E-17
	KRT5	Basal	0.49	< 1.0E-17
	CDH3	Basal	0.49	< 1.0E-17
	KRT6B	Basal	0.47	< 1.0E-17
	KRT14	Basal	0.44	< 1.0E-17
	FGFR3	Luminal	0.38	8.9E-16
	GAPDH	Reference	0.36	1.9E-14
	KRT19	Luminal	0.35	1.8E-13
	KRT1	Basal	0.31	7.6E-11
KRT17	*KRT5*	Basal	0.69	< 1.0E-17
	CDH3	Basal	0.66	< 1.0E-17
	KRT16	Basal	0.65	< 1.0E-17
	KRT6A	Basal	0.62	< 1.0E-17
	KRT14	Basal	0.61	< 1.0E-17
	KRT6C	Basal	0.59	< 1.0E-17
	KRT6B	Basal	0.59	< 1.0E-17
	SLC2A1	Marker	0.57	< 1.0E-17
	FGFR3	Luminal	0.41	< 1.0E-17
	KRT19	Luminal	0.40	< 1.0E-17
	CD44	Basal	0.37	4.4E-15
	KRT1	Basal	0.35	5.6E-14
GPRC5A	*KRT19*	Luminal	0.35	8.8E-14
	ERBB3	Luminal	0.32	6.0E-12

Correlation analysis was conducted to investigate the similarity of gene expression in bladder tumors (TCGA [31]) for the markers identified in this study (*SLC2A1*, *GPRC5A* and *KRT17*), reference genes (*ACTB*, *GAPDH*, and *ALDOB*), and 'basal' and 'luminal' subtype markers Choi *et al.* reported [28]. Only genes correlated with Pearson's $r < 0.3$ and p value $< 1.0 \times 10^{-10}$) are shown. Correlation plot is shown in Supplementary Figure 4.
